# 

**Published:** 1904-11-12

**Authors:** 


### NOTICE TO CORRESPONDENTS.

All MSS., Letters, Books for Review, and other matters intended for the Editor should be addressed THE EDITOR, "THE HOSPITAL," THE HOSPITAL BUILDINGS, 28 & 29 SOUTHAMPTON STREET, STRAND, W.C.

The Editor cannot undertake to return rejected MS., even when accompanied by stamped directed envelope.

**Notice to Advertisers and Subscribers.**

All Advertisements, Orders for Copies of THE HOSPITAL, and Business Communications should be addressed to **THE MANAGER (Not to the Editor),** THE HOSPITAL, 28 & 29 Southampton Street, Strand, London, W.C.

The Cost of Subscription, post free, is as follows:—Per week, 3½d.; per quarter, 3s. 3d.; per half-year, 6s. 6d.; per annum, 13s. Foreign and Colonial:—Per annum, 19s. 6d., payable, in all cases, in advance.

**NOTICE.—Vol. XXXVI. of "The Hospital" (April to September 1904), in handsome cloth binding, is now on sale at the office of this Journal, price 10s. 6d. Cases for binding the Numbers contained in this Volume 2s. Index to Vol. XXXVI., 3½d., post free.**

**The Monthly part for November is now ready. Price 1s., by post, 1s. 3d.**

### BOOKS RECEIVED.

Hazell, Watson, and Viney, Ltd.

"Mediterranean Winter Resorts," 5th Edition. By Eustace A. Reynolds Ball.

G. Philip and Son, Ltd.

"Philip's Handy Volume Atlas of the County of London," 4th Edition.

Simpkin, Marshall and Co., Ltd.

"Health in Infancy." By T. M. Allison, M.D.

J. and A. Churchill.

"Urine Examination Made Easy." By T. Carruthers, M.B., Ch.B.

Cornish Bros., Birmingham.

"The Sterilisation of the Hands." By Chas. Leedham-Green, M.B., F.R.C.S.

The Scientific Press, Limited.

"The Modern Nursing of Consumption." By Dr. Jane Walker.

Baillière, Tindall and Cox.

"The Nutrition of the Infant." By Ralph Vincent, M.D. (2nd Edition).

"Guide to the Examination of the Throat, Nose, and Ear." By W. Lamb, M.D., Edin., M.R.C.P., Lond.

"Malignant Disease of the Larynx." By P. R. W. de Santi, F.R.C S.

"Handbook of Diseases of the Ear." By R. Lake, F.R.C.S.

"After Treatment of Operations." By P. L. Mummery, M.B., F.R.C.S. (2nd Edition.)

### MEDICAL APPOINTMENTS.

H. A. Baber, M.R.C.S.Eng., L.S.A., District Medical Officer of the Tavistock Union. E. M. Brockbank, M.D., M.R.C.P., Junior Physician to the Manchester Children's Hospital. P. S. Buchanan, M.B., etc., in charge of the Midwifery Outdoor Department of the Glasgow Public Dispensary. C. D. Francis Burney, M.R.C.S., L.R.C.P., Divisional Surgeon to the "R" Division, Metropolitan Police (Deptford), and Divisional Surgeon to the Deptford Victualling Yard Section of the Woolwich Division. W. H. Davison, M.B., B.S., Resident Assistant Medical Officer, Aston Union Workhouse. E. G. Gibbs Smith, L.R.C.P., L S.A., D.P.H., Aural Assistant, Throat, Nose, and Ear Department, London Hospital. Aarons S. Jervois, M.D.Edin., M.R.C.P. Lond., has been appointed Pathologist and Curator of the Museum Hospital for Women, Soho Square, W. J. A. Laycock, L.R.C.P.I., L.M., District Medical Officer of the Burnley Union. F. E. Mathews, L.R.C.P., M.R.C.S., District and Workhouse Medical Officer of the Nantwich Union. Charles H. Melland, M.D. Lond., M.R.C.P., Assistant Physician for Children to the Northern Hospital, Manchester. A. Balfour Nairn, L.R.C.P.S.Edin., L.S.P.F.Glasg., Pathologist and Assistant Medical Officer to the West Riding Asylum, Wakefield. T. Roberts, M.B., C.M.Edin., Medical Officer of the Workhouse of the Carnarvon Union. W. E. Rothwell, M.B., B.S.Vict., Junior Resident Medical Officer, Crumpsall Workhouse, Manchester. E. Graham Taylor, M.B., etc., to the Gynæcological Department of the Glasgow Public Dispensary. E. D. Telford, M.A., F.R.C.S., L.R.C.P., Junior Surgeon to the Manchester Children's Hospital and Dispensary. Arthur H. Tovey, L.R.C.P., M.R.C.S., House-Physician to the General Lying-in Hospital, Lambeth, S E. C. H. S. Vinter, M.R.C.S., L.R.C.P., District Medical Officer and Public Vaccinator for the Blackpool South District. A. Landon Whitehouse, M.R.C.S., L.R.C.P., L.D.S.Eng., Junior Demonstrator, Royal Dental Hospital of London.

GLASGOW ROYAL INFIRMARY.—The following appointments have been made for six months:—Resident Physicians: James B. Patterson, L.R.C.P. and S.Glasg., to Dr. McVail; R. C. Blyth, L.R.C.P. and S.Edin., to Dr. Middleton; George H. Wildish, M.B., Ch.B.Glasg., to Dr. Steven; Andrew Connol, M.B., Ch.B.Glasg., to Dr. Munro; E. N. Coutts, M.B., Toronto, L R.C.P. and S.Edin., to Dr. Allan. Resident Surgeons: John B. Stewart, M.B., Ch.B.Glasg. (Senior), to Dr. Barlow; D. H. MacPhail, D.H., M.B., Ch.B.Glasg., to Dr. Clark; J. Roy McVail, M.B, Ch.B.Glasg., to Dr. Knox; George C. Neilson, M.B., Ch.B Glasg., to Dr. Adams; Howard H. Patrick, M.B. Ch.B.Glasg., to Dr. Newman; Charles D. Lochrane, M.B., Ch.B.Edin., to Mr. Pringle; John D. McCallum, M.A., M.B., Ch.B.Glasg., to Dr. McLennan; Hugh H. Fulton, M.B., Ch.B. Glasg., Resident Casualty Surgeon; Anna P. Martin, M.B., Ch.B.Glasg., to Dr. Kelly; Malcolm Hutton, M.A., B.Sc., Ch.B.Glasg., to Dr. Ramsay.

**"The Hospital" Institutional Library.**

**"Hospitals and Asylums of the World." 4 Vols., with a Portfolio of Plans, complete £12 12s.**

**"Burdett's Hospitals and Charities, 1904." 5s. net.**

**"Burdett's Official Nursing Directory." 3s. net.**

**"Cottage Hospitals, General, Fever, and Convalescent." 10s. 6d.**

**"Uniform System of Accounts for Hospitals and Public Institutions." New Edition. 4s. net.**

**"Hospital Expenditure: The Commissariat." 2s. 6d.**

**"A Legal Handbook for the Use of Hospital Authorities." 2s. 6d.**

**"The Cottage Hospital Case Book or Register of Patients." 10s. and 12s. 6d.**

**All these are published by the SCIENTIFIC PRESS, Ltd., and may be obtained through any bookseller, or direct from the publishers, 28 and 29 Southampton Street, Strand, London, W.C.**

**Medical Appointments.**

AN EXAMINATION OF CANDIDATES for entry into the Medical Department of the Royal Navy will be held on 14th November next and following days at Examination Hall, Thames Embankment.

The forms to be filled up by Candidates will be supplied on application to the MEDICAL DIRECTOR-GENERAL.

Admiralty, 18 Victoria Street, S.W.

30th September, 1904.

*Published by*
**THE SCIENTIFIC PRESS, Ltd.,**
28 & 29 Southampton St.,
Strand, London, W.C.

AT 3/6

**NURSING: ITS THEORY AND PRACTICE.**
By Dr PERCY G. LEWIS.

**SURGICAL WARD-WORK AND NURSING.** With particulars of Instruments used in various operations. By ALEXANDER MILES, M.D. **3/10** post free.

**OPHTHALMIC NURSING.**
By SYDNEY STEPHENSON, M.B **3/10** post free.

**DIET IN SICKNESS AND IN HEALTH.**
By Mrs. ERNEST HART.

**OUTLINES OF INSANITY.**
By FRANCIS H WALMESLEY, M.D Popular Edition **1/6**.

**FIRST AID TO THE INJURED AND MANAGEMENT OF THE SICK.** By E J. LAWLESS, M.D.

AT 2/6

**ELEMENTARY ANATOMY & SURGERY FOR NURSES.**
By WM. MCADAM ECCLES M.S., M B.

**HOSPITAL SISTERS AND THEIR DUTIES.**
By EVA C. E. LÜCKES.

**NURSING IN DISEASES OF THE THROAT, NOSE, AND EAR.** By P. MACLEOD YEARSLEY, F.R.C.S.

**MEDICAL GYMNASTICS: Including the Schott (Nauheim) Movements.** By AXEL V. GRAFSTROM, M.D.

**SPINAL CURVATURE AND AWKWARD DEPORTMENT.**
By S. G. MULLER English Edition by RICHARD GREENE, F.R.C.P.

AT 1/-

**HOSPITAL AND PRIVATE NURSING.**

By Miss S. E ORME.

**FEVERS AND INFECTIOUS DISEASES.**
By WM. HARDING, M.D.

**MIDWIVES' POCKET BOOK.**

By HONNOR MORTEN.

**GYNÆCOLOGICAL NURSING.**
By HAWKINS AMBLER, F.R.C.S.

**NOTES ON PHARMACY & DISPENSING FOR NURSES.**

By C. J. S. THOMSON.

**THE MODERN NURSING OF CONSUMPTION.**
By Dr. JANE WALKER **1/2** post free.

**THE EXAMINATION OF THE URINE.**
By Dr. J. K. WATSON. **1/1** post free.

AT 6d.

**ON PREPARATION FOR OPERATION IN PRIVATE HOUSES.** By STANMORE BISHOP, F.R.C.S.

**MODERN NURSING IN PRIVATE PRACTICE.**
By Sir WILLIAM BENNETT. **7d.** post free.

**SCHOTT TREATMENT FOR CHRONIC HEART DISEASES.**
By RICHARD GREENE, F.R.C.P.

**POCKET BOOK OF TEMPERATURE CHARTS.**
Containing 17 Twelve-Hour and 8 Four-Hour Charts. **7d.** post free.

**"SIMPLEX" DISTRICT NURSES' CASE-BOOK.**
Containing space for 50 cases, ruled and printed. **7d.** post free.

NEW CATALOGUE OF NURSING MANUALS POST FREE ON APPLICATION.

**THE SCIENTIFIC PRESS, LTD.,** Publishers and Booksellers of all kinds of Nursing Text-books, Case-books, Charts, &c.
**28 & 29 Southampton St., Strand, London, W.C.**

